# Feasibility of Outpatient Transforaminal Lumbar Interbody Fusion (TLIF) in Chronic Inflammatory Demyelinating Polyneuropathy: A Case Report

**DOI:** 10.7759/cureus.90517

**Published:** 2025-08-19

**Authors:** Miguel Coimbra, Sara Fernandes, Ana Sousa, Joana Cortesão, Marta Azenha

**Affiliations:** 1 Anesthesiology, Unidade Local de Saúde de Coimbra, Coimbra, PRT

**Keywords:** chronic inflammatory demyelinating polyneuropathy (cidp), multimodal analgesia, neuromuscular diseases, outpatient anesthesia, total intravenous anesthesia (tiva)

## Abstract

Chronic inflammatory demyelinating polyneuropathy (CIDP) is a rare autoimmune neuropathy characterized by progressive motor and sensory dysfunction. Anesthetic management is challenging due to increased sensitivity to neuromuscular blockers, heightened pain perception, and potential postoperative respiratory compromise. There is limited evidence on the feasibility of outpatient surgery in CIDP patients. We present the case of a 62-year-old male with stable CIDP who underwent minimally invasive transforaminal lumbar interbody fusion (TLIF) as a day-case procedure. Anesthetic management included total intravenous anesthesia (TIVA) without neuromuscular blockade, careful intra- and postoperative monitoring, and multimodal analgesia. The patient experienced no complications and was safely discharged within 24 hours. This report supports the potential feasibility of outpatient spinal surgery in carefully selected CIDP patients, highlighting the importance of multidisciplinary planning and institutional capacity for safe perioperative care.

## Introduction

Chronic inflammatory demyelinating polyneuropathy (CIDP) is an acquired autoimmune neuropathy marked by progressive or relapsing sensorimotor dysfunction, with an estimated prevalence ranging from one to nine per 100,000 individuals, although misdiagnosis remains common [1]. The pathophysiology involves immune-mediated segmental demyelination of peripheral nerves, often accompanied by secondary axonal degeneration. These changes impair saltatory conduction, resulting in reduced conduction velocities, conduction block, and motor and sensory deficits. Both cellular and humoral immune mechanisms are involved, notably macrophage-mediated myelin stripping and autoantibody activity against peripheral nerve antigens. Clinically, CIDP typically presents with symmetrical weakness, sensory loss, and areflexia and may occasionally involve autonomic dysfunction [2].

Treatment includes corticosteroids, immunosuppressive therapy, intravenous immunoglobulin (IVIG), and plasma exchange [3]. Anesthetic considerations in CIDP patients include increased sensitivity to neuromuscular blockers (NMBAs), heightened neuropathic pain perception, and potential respiratory compromise due to neuromuscular weakness [4,5]. Additionally, while CIDP is not classically associated with malignant hyperthermia (MH), its overlap with other neuromuscular disorders justifies caution in anesthetic selection [6]. While outpatient surgery is increasingly performed, its safety and efficacy in CIDP patients remain inadequately documented [7]. We report the case of a patient with stable CIDP who successfully underwent outpatient minimally invasive transforaminal lumbar interbody fusion (TLIF). This case highlights the anesthetic considerations and perioperative strategies that supported a safe and uneventful recovery.

## Case presentation

A 62-year-old male (60 kg, 162 cm; BMI: 22.9 kg/m²) diagnosed with CIDP in 2011 presented with chronic lumbar radiculopathy due to L4-L5 spondylolisthesis and disc herniation. His medical history included type 2 diabetes mellitus, hypertension, past smoking, and previous hospitalization for pneumonia (2014). CIDP management included azathioprine, prednisolone, and regular plasma exchange every three weeks. At the time of surgery, the patient was clinically stable, with muscle strength graded 5 in all limbs by the Medical Research Council (MRC) scale [8], except for mild weakness (grade 4+) in the left-hand finger abduction, generalized areflexia, reduced lower-limb vibration sense, and mild muscle atrophy. Ambulation was independent but impaired by lumbar pain. When plasma exchange sessions were delayed, he typically experienced worsening sensory symptoms, motor weakness, and balance impairment; the last session was performed one week preoperatively. Neurology evaluation confirmed stable disease without contraindications for surgery. Preoperative evaluation (American Society of Anesthesiologists (ASA) III) demonstrated normal blood tests, chest radiograph, and ECG. Respiratory function tests were not performed, although their use could have been considered. The procedure took place at a facility with institutional capacity for overnight stay. Chronic medications, including azathioprine and prednisolone, were continued perioperatively. Given the low chronic dose of prednisolone (7.5 mg/day), current guidelines do not support routine steroid supplementation in the absence of adrenal insufficiency [9]. Anesthesia was induced and maintained with total intravenous anesthesia (TIVA) using propofol and remifentanil. In the absence of neuromuscular blockers, higher effect-site target concentrations of propofol and remifentanil were selected to ensure adequate intubating conditions and immobility. Lidocaine (1.5 mg/kg) was administered to improve intubation conditions and suppress airway reflexes. Monitoring included ECG, pulse oximetry, noninvasive blood pressure, capnography, oropharyngeal temperature, bispectral index (BIS), and blood glucose. Intubation was performed using a Macintosh laryngoscope with a reinforced 7.5-mm endotracheal tube, followed by prone positioning. Intraoperative multimodal analgesia included intravenous paracetamol, ketorolac, tramadol, and wound infiltration with ropivacaine 0.375%. Antiemetic prophylaxis included dexamethasone 4 mg and ondansetron 4 mg. Surgical duration was 98 minutes; total anesthesia time was 160 minutes without complications. Postoperatively, in the post-anesthesia care unit (PACU), pain management continued with paracetamol and ketorolac, without the need for additional opioids. Pain scores peaked at 4 (Numeric Rating Scale) [10] and decreased to 0 at discharge. Clinical and neurological evaluations remained stable, allowing discharge in less than 24 hours. Follow-up at one week later confirmed symptom improvement without analgesic requirements or neurological deterioration.

## Discussion

CIDP involves segmental demyelination of peripheral nerves and possible secondary axonal loss, leading to motor, sensory, and autonomic dysfunction [2,3]. The anesthetic management of CIDP patients requires careful planning due to the potential for prolonged neuromuscular blockade, heightened pain sensitivity, respiratory compromise, and theoretical malignant hyperthermia (MH) risk.

Various anesthetic techniques have been reported in patients with CIDP, including general anesthesia, neuraxial anesthesia, and peripheral nerve blocks. Neuraxial anesthesia has been described in case reports with uneventful outcomes [11], but potential risks, such as unpredictable block spread, local anesthetic neurotoxicity in demyelinated fibers, and theoretical neurological worsening, are largely extrapolated from data in multiple sclerosis and other demyelinating neuropathies, given the scarcity of CIDP-specific evidence. High sensory levels during neuraxial anesthesia may impair intercostal muscle function, increasing the risk of respiratory compromise. Peripheral nerve blocks have also been reported [12], although altered peripheral conduction could influence onset time, block density, and duration. In our case, general anesthesia was considered the most appropriate choice, providing airway protection, stable surgical conditions, and avoiding the administration of local anesthetics into potentially vulnerable neural structures.

The autoimmune-mediated demyelination characteristic of CIDP significantly enhances sensitivity to non-depolarizing NMBAs, potentially leading to prolonged blockade and delayed recovery [4,13]. Mortenson et al. reported prolonged and unpredictable NMBA responses in CIDP patients, strongly suggesting cautious use or complete avoidance of these agents [5]. Our approach of utilizing TIVA without NMBAs aligns with current recommendations. However, when clinically necessary, sugammadex provides rapid and complete NMBA reversal, significantly mitigating postoperative respiratory risks [14,15].

Neuropathic pain in CIDP arises from peripheral nerve injury and central sensitization mechanisms, resulting in increased postoperative analgesic requirements [16]. Multimodal analgesia in our patient provided effective pain control without opioid escalation, facilitating early ambulation and discharge. Respiratory involvement, including diaphragmatic dysfunction, may be subclinical in CIDP but can be exacerbated by anesthesia or positioning [17,18]. While our patient had no overt signs of pulmonary disease, preoperative respiratory function testing might have added reassurance [19]. Continuous postoperative respiratory observation supported early identification of potential compromise. Although CIDP is not directly linked to MH, overlapping characteristics with other neuromuscular conditions warrant precaution in anesthetic selection. The use of propofol-based TIVA and the avoidance of neuromuscular blockers effectively eliminated potential MH triggers, enhancing safety in this population [6].

Outpatient spine surgery in patients with neuromuscular disorders like CIDP is uncommon and requires meticulous patient selection, disease stability, multidisciplinary collaboration, and institutional resources for overnight monitoring if necessary [7]. The minimally invasive surgical approach employed significantly contributed to reduced tissue trauma, less postoperative pain, and faster recovery, enabling safe early discharge. This successful outcome, however, should not generalize outpatient spinal procedures for all CIDP patients; individualized assessments and institutional readiness remain critical. Prospective studies and multicentric registries are needed to clarify safety guidelines and perioperative management strategies for CIDP patients undergoing ambulatory spinal procedures.

## Conclusions

Outpatient TLIF using TIVA without neuromuscular blockers was safely performed in a patient with stable CIDP. Key factors contributing to the successful outcome included careful patient selection, multidisciplinary planning, multimodal analgesia, minimally invasive surgical technique, and institutional capacity for extended monitoring. This case contributes to the limited literature on perioperative care in CIDP and supports the cautious expansion of outpatient surgery in select patients with neuromuscular disorders.
